# Green-synthesized quantum dots from quercus brantii for infected polymicrobial wound healing: mechanisms and biocompatibility

**DOI:** 10.1186/s12896-026-01183-5

**Published:** 2026-06-16

**Authors:** Yasaman Sadat Nabipour, Ardeshir Hesampour, Salman Ahmady Asbchin, Maryam Tajabadi, Arman Rostamzad

**Affiliations:** 1 Department of Biology, CT.C., Islamic Azad University, Tehran, Iran; 2 Department of Microbiology, Faculty of Sciences, University of Mazandaran, Babolsar, Iran; 3 Department of Biology, Faculty of Sciences, Ilam University, Ilam, Iran

**Keywords:** Green synthesis, Silver quantum dots, Quantum confinement, Polymicrobial infection, Biofilm, Wound healing, Cytotoxicity, Reactive oxygen species

## Abstract

**Background:**

The rise of antimicrobial resistance (AMR) has created an urgent need for alternative therapies, particularly against biofilm-driven polymicrobial wound infections. Although green-synthesized nanoparticles have emerged as promising candidates, many studies lack rigorous validation of their quantum properties, fail to clarify mechanisms of action, and omit essential cytotoxicity profiling in human cells.

**Objective:**

To synthesize silver (Ag), copper (Cu), and zinc oxide (ZnO) quantum dots (QDs) using *Quercus brantii* acorn extract, confirm quantum confinement effects, and evaluate their antimicrobial efficacy, mechanistic basis, and biosafety in a clinically relevant murine wound model.

**Methods:**

QDs were synthesized via a standardized hydrothermal approach. Quantum confinement was verified using photoluminescence (PL) spectroscopy and Tauc plot analysis. Antimicrobial activity was tested against multidrug-resistant (MDR) clinical isolates of *Pseudomonasaeruginosa*, methicillin-resistant *Staphylococcus aureus* (MRSA), *Acinetobacter baumannii*, and *Klebsiella pneumoniae*. To differentiate oxidative stress from chemical artifacts, we employed non-thiol reactive oxygen species (ROS) scavengers (Trolox, Mannitol) and electron paramagnetic resonance (EPR). Cytotoxicity was assessed in human keratinocytes (HaCaT) and dermal fibroblasts (HDF). In vivo efficacy was evaluated in a murine excisional wound model infected with a polymicrobial consortium, tracking pathogen-specific clearance. Hydrogel rheology and stability were also characterized.

**Results:**

Monodispersed, crystalline QDs with evidence consistent with quantum confinement were obtained (Ag-QDs: 7.2 ± 1.5 nm; apparent bandgap: 2.85 eV). Ag-QDs showed strong antimicrobial activity (MIC: 4.5–18.1 µg/mL) and biofilm inhibition (up to 85% at ½×MIC). Mechanistic experiments, including EPR and ROS-scavenger assays, supported ROS-associated oxidative stress as a major contributor to bacterial killing under the tested conditions. Ag-QDs exhibited bactericidal activity at concentrations that were non-toxic to human skin cells in the employed assay (Selectivity Index for MRSA: 18.9). In vivo, topical application of an Ag-QD hydrogel (shear-thinning) accelerated wound closure to 95.3% by Day 14 (comparable to uninfected controls) and reduced the total bacterial burden by 4.2 log₁₀ CFU/g; all four pathogens were below the detection limit at the study endpoint. No overt systemic toxicity was observed based on the assessed biomarkers. Resistance development in serial passage assays was limited (2-fold MIC increase after 30 passages), noting that longer-term polymicrobial studies are required to fully evaluate resistance evolution.

**Conclusion:**

*Q. brantii*-mediated Ag-QDs are a promising green-synthesized nanomaterial with physicochemical characteristics consistent with quantum-dot behavior and notable antimicrobial and antibiofilm activity against MDR polymicrobial communities. In a preclinical murine wound model, topical Ag-QD hydrogel treatment was associated with accelerated wound closure and marked reductions in bacterial burden, alongside a favorable preliminary biosafety profile based on the endpoints assessed. Further studies are warranted to validate efficacy in clinically relevant chronic wound settings (e.g., diabetic models), to expand long-term toxicology, and to clarify resistance evolution and mechanistic pathways in polymicrobial systems.

## Introduction

Chronic wound infections impose a substantial global health burden, affecting millions of patients and generating annual healthcare costs exceeding 28 billion in the United States alone [1]. More than 80% of chronic wounds harbor polymicrobial biofilms, which confer up to 1,000-fold increased tolerance to conventional antibiotics and drive the persistence of multidrug-resistant (MDR) organisms such as methicillin-resistant *Staphylococcus aureus* (MRSA) and carbapenem-resistant *Acinetobacter baumannii* [2, 3]. The limited efficacy of existing therapies underscores an urgent need for novel antimicrobial strategies capable of disrupting biofilm architecture and overcoming resistance mechanisms.

Nanotechnology offers a transformative approach to this challenge. Quantum dots (QDs)—semiconductor or metallic nanoparticles typically below 10 nm in size—exhibit unique physicochemical properties arising from quantum confinement, including size-tunable bandgaps, discrete electronic states, and enhanced catalytic activity [4, 5]. Unlike bulk materials or larger nanoparticles, true QDs display distinct optical signatures such as size-dependent photoluminescence. Metallic QDs, particularly those composed of silver (Ag), copper (Cu), and zinc oxide (ZnO), have demonstrated broad-spectrum antimicrobial effects through multimodal mechanisms, including membrane disruption, protein dysfunction, DNA damage, and the generation of reactive oxygen species (ROS) [6, 7]. This multi-target activity is thought to reduce the likelihood of resistance development [8].

Despite these advantages, the field of green synthesis is hindered by methodological inconsistencies. Many studies inappropriately classify ultra-small nanoparticles as “quantum dots” without providing evidence of quantum confinement, such as photoluminescence spectra or widened bandgaps [5]. Furthermore, mechanistic investigations frequently rely on thiol-containing ROS scavengers like N-acetylcysteine (NAC), which can chemically sequester silver ions (Ag⁺) independently of ROS quenching, leading to artifactual conclusions [9]. Equally concerning, numerous translational studies bypass critical in vitro cytotoxicity assessments in human cell lines, proceeding directly to animal models and thereby overlooking potential dermal toxicity [6].

*Quercus brantii* (oak) acorns are rich in hydrolyzable tannins and flavonoids, compounds known for their reducing and capping capabilities [10]. Although oak extracts have been traditionally used for wound healing, their potential to generate rigorously characterized metallic QDs with verified quantum effects and documented human safety profiles remains unexplored.

This study addresses these critical gaps with the following objectives: (1) synthesize Ag, Cu, and ZnO QDs using *Q. brantii* extract and definitively confirm quantum confinement via photoluminescence and bandgap analysis; (2) evaluate antimicrobial efficacy against a panel of MDR wound pathogens while rigorously controlling for residual phytochemicals; (3) elucidate the mechanism of action using non-thiol ROS scavengers and electron paramagnetic resonance (EPR) to ensure mechanistic specificity; (4) assess cytotoxicity in human keratinocytes (HaCaT) and dermal fibroblasts (HDF) to establish a therapeutic window; and (5) validate therapeutic potential in a stringent murine polymicrobial wound model, tracking individual pathogen clearance and tissue regeneration. A graphical overview of the study design and key findings is presented in Fig. 1.

**Fig. 1:**
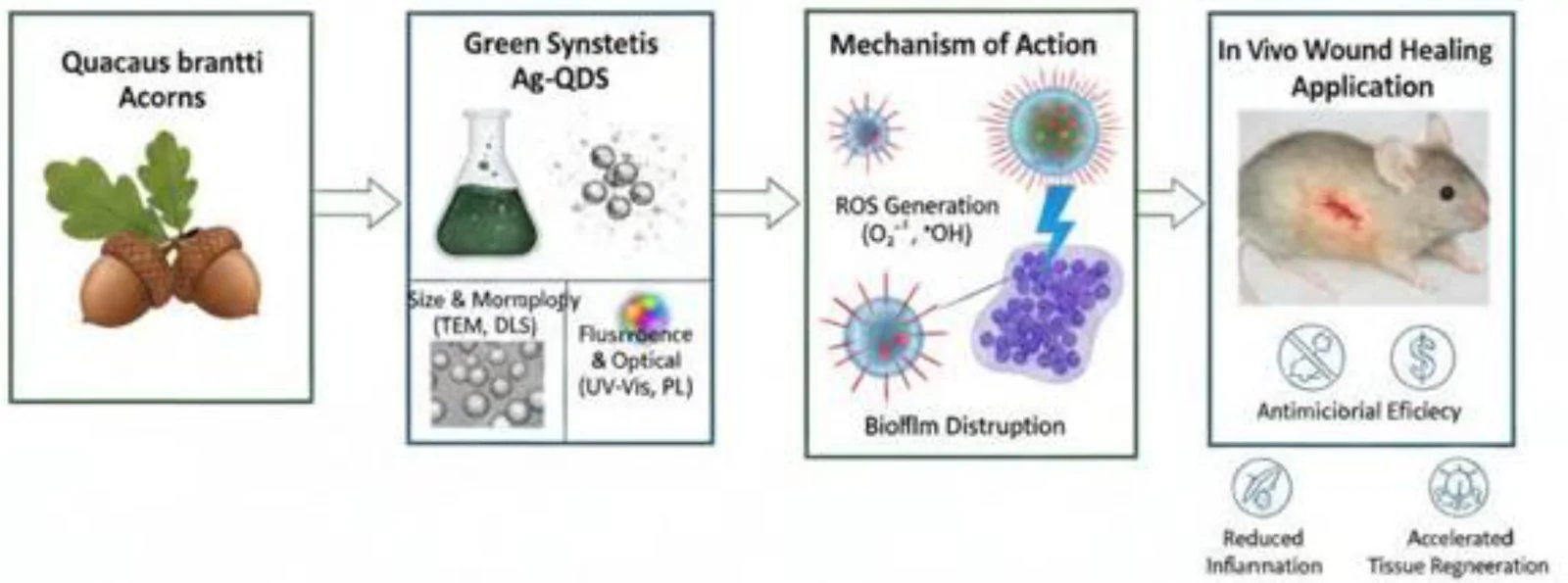
Graphical representation of the study. Green synthesis of Quercus brantii-mediated silver quantum dots (Ag-QDs) and their multi-targeted therapeutic application against polymicrobial biofilm-infected wounds

## Materials and methods

### Phytochemical profiling and extract standardization

Mature acorns of *Quercus brantii* were collected from western Iran (33.45°N, 48.50°E) in September 2023 and authenticated (Voucher No. QB-AC-2023-01). An aqueous extract was prepared by refluxing 50 g of powdered kernels in 1 L of deionized water at 80 °C for 2 h, followed by filtration (0.22 μm) and lyophilization.

Phytochemical analysis was conducted using LC-MS/MS (Agilent 6460 Triple Quad) to definitively identify major constituents. Separation was achieved on a Zorbax Eclipse Plus C18 column (2.1 × 50 mm, 1.8 μm) with a gradient elution of water (0.1% formic acid) and acetonitrile at 0.3 mL/min. Detection was performed in negative ion mode with electrospray ionization (ESI). Compounds were identified by comparing retention times and mass spectra with authentic standards (gallic acid, ellagic acid, methyl gallate, quercetin-3-O-glucoside, kaempferol-3-O-rhamnoside; Sigma-Aldrich). Total phenolic and flavonoid contents were determined using Folin-Ciocalteu and aluminum chloride methods, respectively. All analyses were performed in triplicate.

### Green synthesis and verification of quantum confinement

#### Synthesis

Ag-QDs, Cu-QDs, and ZnO-QDs were synthesized by reacting 10 mL of standardized oak extract with 90 mL of 1 mM AgNO₃, 2 mM CuCl₂, or 3 mM zinc acetate (pH adjusted to 10.0 ± 0.2 with 1 M NaOH), respectively, under controlled thermal conditions (60–120 °C). Purification involved triple centrifugation (30,000 × g) and washing to remove unbound phytochemicals. Of note, synthesis conditions varied by QD type due to differing chemical requirements, a factor to consider when comparing properties directly.

#### Verification of quantum properties

To distinguish QDs from conventional nanoparticles, we employed:

- **Photoluminescence (PL) Spectroscopy**: Emission spectra were recorded at various excitation wavelengths (Horiba FluoroMax-4) to identify size-dependent fluorescence.
- **Tauc Plot Analysis**: Optical bandgaps (Eg) were calculated from UV-Vis absorption data using the relation (αhν)^n = A(hν – Eg), where *n* = 1/2 for direct bandgap semiconductors. A widened bandgap relative to bulk material confirms quantum confinement.
- **HR-TEM and XRD**: Crystallinity and size distribution (< 10 nm) were confirmed as prerequisite criteria.

#### Quantum confinemen

To exclusively validate the contribution of the quantum confinement effect to the observed biological performance, a non-quantum silver nanoparticle control (Ag-NPs-Control) was synthesized using the same Quercus brantii extract. This yielded standard nanoparticles with a mean diameter of 45 pm 5 nm, which is well above the quantum confinement threshold of approx 10 nm. Surface chemistry remained consistent between the two groups, as evidenced by comparable zeta-potential values (-24.3 mV for Ag-AQDs vs. -23.8 mV for Ag-NPs-Control, *p* < 0.05).

#### Preparation of hydrogel

Preparation of Hydrogel: The synthesized Ag-QDs, Cu-QDs, and ZnO-QDs were each homogeneously dispersed into a Carbopol 940 hydrogel matrix (1% w/v, neutralized to pH 6.5 ± 0.2 with triethanolamine) at a final QD concentration of 0.5% w/w (5 mg/g of gel). This concentration was rationally selected based on the in vitro MIC values and cytotoxicity data (CC₅₀ > 85 µg/mL on HDFs) to ensure sustained therapeutic antibacterial activity at the wound site without inducing localized tissue toxicity. To confirm the baseline biocompatibility and lack of inherent cytotoxicity of the delivery vehicle, the blank Carbopol hydrogel (without QDs) was evaluated independently. Furthermore, a commercial Silver Sulfadiazine (SSD) cream at the standard clinical concentration of 1% w/w was utilized as the positive control in all comparative in vivo and in vitro assays.

#### In vitro drug release study

The in vitro release profile of Ag-QDs from the Carbopol hydrogel matrix was evaluated using the dialysis bag method. Briefly, 1 g of the Ag-QD-loaded gel was placed in a dialysis bag (MWCO 12–14 kDa) and immersed in 50 mL of phosphate-buffered saline (PBS, pH 7.4) at 37 °C under constant magnetic stirring (100 rpm). At predetermined time intervals (1, 2, 4, 6, 8, 12, 24, 48, and 72 h), 1 mL aliquots were withdrawn and immediately replaced with an equal volume of fresh PBS to maintain sink conditions. The concentration of released active agent was determined using UV-Vis spectrophotometry at 420 nm. The cumulative release percentage was calculated and plotted as a function of time. All experiments were performed in triplicate.

### Physicochemical characterization

Comprehensive characterization included UV-Vis spectroscopy, HR-TEM (JEOL JEM-2100 F), XRD (Bruker D8), FTIR (Thermo Nicolet), XPS (Kratos AXIS Supra), and DLS/zeta potential measurement (Malvern Zetasizer). Residual free metal ions were quantified by ICP-MS after ultrafiltration (3 kDa cutoff).

#### Stability in simulated wound fluid (SWF)

QDs were incubated in SWF containing 4% bovine serum albumin (BSA) for 24 h. FTIR and XPS were repeated post-incubation to assess stability of the phytochemical corona against protein displacement (Vroman effect).

#### Nanoparticle characterization

To evaluate the precise morphology, primary core size, and distribution of the synthesized quantum dots (Carbon, Ag, Cu, and ZnQ QDs), high-resolution transmission electron microscopy (HR-TEM) was employed. The samples were prepared by drop-casting a diluted suspension of the QDs onto carbon-coated copper grids and allowed to dry at room temperature prior to imaging. Furthermore, the optical properties and successful synthesis of the QDs were analyzed using a UV-Vis spectrophotometer, with absorption spectra recorded across the relevant wavelength ranges to identify specific characteristic peaks.

### Microbial strains and culture conditions

MDR clinical isolates (*P. aeruginosa*, MRSA, *A. baumannii*, *K. pneumoniae*) were obtained from tertiary care hospitals (Ethical Approval: IR.MUI.REC.1401.215 of Ilam University) and authenticated via MALDI-TOF MS. Resistance profiles were confirmed per CLSI guidelines [13].

### In vitro antimicrobial evaluation

#### MIC/MBC determination

Broth microdilution assays were performed with two-fold serial dilutions of QDs (500–3.9 µg/mL) according to established guidelines [14]. A critical control group included oak extract alone at concentrations equivalent to those present in QD suspensions (determined gravimetrically with an estimated error of ± 5%).

#### Biofilm assays

Biofilm inhibition and eradication were assessed using crystal violet staining and confocal laser scanning microscopy (CLSM) with Live/Dead BacLight staining.

#### Synergy testing

Checkerboard assays evaluated synergy between Ag-QDs and conventional antibiotics (ciprofloxacin, vancomycin).

#### Determination of synergistic effect (FIC Index)

The fractional inhibitory concentration (FIC) index was calculated to quantitatively evaluate the synergistic interaction between the phytochemical corona and the silver core. The MIC values of bio-synthesized Ag-QDs, chemically synthesized bare Ag-NPs (prepared via standard citrate reduction without plant extract), and pure Quercus brantii extract against S. aureus were determined using the broth microdilution method. The FIC index was calculated as:

$$\eqalign{ {\rm{FIC}} = & {\rm{}}\left( {{\rm{MIC}}\,{\rm{of}}\,{\rm{Ag}} - {\rm{QDs}}/{\rm{MIC}}\,{\rm{of}}\,{\rm{bare}}\,{\rm{Ag}} - {\rm{NPs}}} \right){\rm{}} \cr & + {\rm{}}\left( {{\rm{MIC}}\,{\rm{of}}\,{\rm{Ag}} - {\rm{QDs}}/{\rm{MIC}}\,{\rm{of}}\,{\rm{extract}}} \right) \cr}$$

An FIC index ≤ 0.5 indicates synergy, 0.5 < FIC ≤ 1 indicates additivity, and FIC > 1 indicates antagonism.

### Mechanistic investigations: ROS and gene expression

#### ROS detection with specific scavengers

Intracellular ROS was measured using DCFH-DA. To ensure mechanistic specificity, experiments included:

- **N-acetylcysteine (NAC)**: To observe total inhibition (potential chemical + biological effect).
- **Trolox** (water-soluble vitamin E analog) and **Mannitol**: Non-thiol scavengers specific to general ROS and hydroxyl radicals, respectively, to decouple chemical precipitation of silver from ROS quenching.

#### Electron paramagnetic resonance (EPR)

Direct detection of ROS species (superoxide anion O₂•⁻ and hydroxyl radical •OH) was performed using spin traps (DMPO and TEMPO) on a Bruker EMXplus spectrometer, providing artifact-free validation.

#### qRT-PCR

Expression of biofilm-related genes (*icaA*, *bap*, *lecA*, *mrkA*) was quantified using validated housekeeping genes (*rpoB*, 16 S rRNA) normalized via NormFinder. Primer sequences and efficiencies are provided in Table 5-A1 (Supplementary Information).

To ensure the reliability of the gene expression data, the stability of the reference genes was evaluated using the NormFinder algorithm. The [*16 S rRNA*] gene exhibited the highest stability value (stability value = 0.015) and was thus selected for the accurate normalization of the target genes.

### Cytotoxicity assessment on human skin cells

To evaluate translational safety, cytotoxicity was assessed in human keratinocytes (HaCaT) and human dermal fibroblasts (HDF).

#### Cell culture

Cells were cultured in DMEM supplemented with 10% FBS at 37 °C, 5% CO₂.

#### MTT assay

Cells were exposed to QD concentrations ranging from 1 to 100 µg/mL for 24 and 48 h. Viability was measured via MTT reduction. CC₅₀ values (concentration reducing viability by 50%) were calculated by non-linear regression.

#### Cytocompatibility of blank carbopol hydrogel

To definitively rule out any background biological interference from the delivery vehicle, the in vitro cytocompatibility of the blank Carbopol hydrogel (without QDs) was evaluated on human dermal fibroblasts (HDFs) using the MTT assay. HDFs were exposed to the blank hydrogel extract (prepared according to ISO 10993 12) for 24 h, and cell viability was measured relative to untreated controls.

#### Intracellular ROS in mammalian cells

ROS levels in HaCaT cells were measured using DCFH-DA to ensure therapeutic doses do not induce oxidative stress in host tissues.

#### Selectivity index (SI)

Calculated as CC₅₀ (HaCaT) / MIC (pathogen). An SI > 10 indicates a favorable safety margin.

### In vivo preclinical validation

All in vivo procedures in this study were conducted in accordance with the National Institutes of Health (NIH) *Guide for the Care and Use of Laboratory Animals*, the national guidelines of Iran for the ethical use of animals in research, and were approved by the Ethics Committee of Ilam University of Medical Sciences (approval code: IR.ILAM.REC.1401.008). As the study did not involve human participants or human-derived materials, the provisions of the latest revision of the Declaration of Helsinki were not directly applicable; however, the general principles of ethical research conduct and respect for animal welfare were strictly followed throughout the study.

Female BALB/c mice (8–10 weeks of age, 18–22 g body weight) were obtained from the Laboratory Animal Breeding and Maintenance Center, Razi School of Pharmacy, Kermanshah, Iran. Upon arrival, animals were acclimatized for at least one week and were housed in the Veterinary Pathobiology Research Center animal facility at Ilam University under standard specific-pathogenfree (SPF) conditions. Mice were kept in polycarbonate cages with corncob bedding, maintained at a temperature of 22 ± 2 °C, relative humidity of 50–60%, and a 12 h light/12 h dark cycle, with free access to standard laboratory chow and water ad libitum. All efforts were made to minimize animal stress and discomfort throughout the study.

All surgical procedures were performed under general anesthesia. Anesthesia was induced by intraperitoneal injection of ketamine (80 mg/kg) and xylazine (10 mg/kg). The depth of anesthesia was assessed by monitoring the pedal withdrawal reflex and observing respiratory rate and pattern. Throughout the procedure, animals were placed on a warming pad to maintain body temperature, and their clinical condition (respiration, mucous membrane color, and response to manipulation) was monitored continuously.

Additional doses of anesthetic agents were administered if required to maintain an adequate depth of anesthesia, and no surgical manipulations were performed in animals exhibiting any signs of inadequate anesthesia. Revised text (Materials and Methods – Euthanasia and humane endpoints): At the end of the experimental period, or when predefined humane endpoints were reached, animals were humanely euthanized in accordance with institutional and national guidelines. Euthanasia was performed under deep anesthesia, induced as described above, followed by cervical dislocation as a secondary physical method to ensure death. Death was confirmed by the absence of heartbeat and respiratory movements and by the loss of corneal reflex. All efforts were made to minimize pain, suffering, and the number of animals used, in line with the 3Rs (replacement, reduction, refinement) principles.

Full-thickness excisional wounds (6 mm) were created on mice (*n* = 144 total) and inoculated with a consortium of the four MDR pathogens (1 × 10⁸ CFU/wound). An uninfected control group (*n* = 18) received sterile PBS. After 24 h, a separate cohort of 6 mice was sacrificed to determine baseline bacterial load (Day 0).

*Treatment Groups* (*n* = 6 per group per time point; separate cohorts for days 7, 14, 21):

(1) Uninfected Control, (2) Infected Untreated, (3) Vehicle (Carbopol gel), (4) Silver Sulfadiazine (SSD), (5) Ag-QD Hydrogel, (6) Cu-QD Hydrogel, (7) ZnO-QD Hydrogel.

The positive control group received a commercial 1% w/w Silver Sulfadiazine (SSD) cream, applied topically at the same frequency and volume as the QD hydrogels.

#### Hydrogel rheology

Shear-thinning behavior was characterized using a rotational rheometer (Anton Paar MCR 302) with cone-plate geometry (40 mm, 1° cone angle) at 25 °C. Viscosity was measured as a function of shear rate (0.1–100 s⁻¹).

#### Hydrogel stability

Formulations were stored at 4 °C, 25 °C, and 40 °C for 90 days. At days 0, 30, 60, and 90, samples were analyzed for pH, viscosity, hydrodynamic diameter (DLS), and antimicrobial activity (MIC against *S. aureus*). Results are summarized in Table 5-A2 (Supplementary Information).

#### In vivo antibacterial assessment (Early Time Points and Terminal Clearance)

To capture the early kinetics of in vivo antimicrobial activity, additional cohorts of Ag-QD-treated mice (*n* = 6 per time point) were sacrificed at Days 1 and 3 post-treatment. Wound tissues were homogenized, serially diluted, and plated on selective media as described previously to quantify total and pathogen-specific bacterial burden (CFU/g tissue). These data, combined with baseline (Day 0) and terminal (Day 14) counts, provide a complete time-course of bacterial clearance.

- **Wound Closure**: Digital planimetry.
- **Pathogen-Specific Burden**: Homogenized tissues were plated on selective media (cetrimide agar for *P. aeruginosa*, mannitol salt agar for *S. aureus*, MacConkey agar for *K. pneumoniae*, and Leeds Acinetobacter Medium for *A. baumannii*) to quantify individual CFU/g, ensuring polymicrobial integrity throughout the study.
- **Histopathology and IHC**: H&E, Masson’s trichrome, and staining for CD31, TGF-β1, and MMP-9.
- **Biodistribution and Toxicity**: ICP-MS analysis of organs and serum biochemistry (ALT, AST, BUN, creatinine).

To verify the establishment and stability of the polymicrobial infection, the microbial burden and species ratios were evaluated at baseline (Day 0, immediately post-infection) and at Day 3 post-infection. The Colony Forming Units (CFU) were quantified from the wound beds, and the relative ratios of the bacterial species were analyzed to assess potential competitive exclusion during the acute infection and early biofilm formation phase.

### Assessment of oxidative stress markers in wound tissue

To validate the in vivo relevance of the ROS-mediated antibacterial mechanism, oxidative stress markers were quantified in wound tissue homogenates. At Days 3, 7, and 14 post-treatment, wound tissues were excised, homogenized in cold PBS (pH 7.4), and centrifuged. The supernatant was used for the following assays: (1) Malondialdehyde (MDA) levels, measured using the thiobarbituric acid reactive substances (TBARS) assay; (2) Superoxide dismutase (SOD) activity, determined via a commercial colorimetric kit; (3) Catalase (CAT) activity, measured using a commercial assay kit. All procedures followed the manufacturers’ protocols, and results were normalized to total protein content.

### Cytokine quantification in wound tissue

To characterize the inflammatory microenvironment, levels of key pro-inflammatory (TNF-α, IL-1β, IL-6) and anti-inflammatory (IL-10) cytokines were measured in wound tissue homogenates at Days 3, 7, and 14. Tissues were homogenized in RIPA buffer containing protease inhibitors, centrifuged, and the supernatant was analyzed using commercial sandwich ELISA kits according to the manufacturers’ instructions. Cytokine concentrations were expressed as pg/mg of total protein.

### Statistical analysis

Data normality was assessed using the Shapiro–Wilk test. Comparisons were made using one-way or two-way ANOVA with Tukey’s post-hoc test. Significance was set at *p* < 0.05. All experiments were performed in triplicate across three independent runs. Outliers were identified using Grubbs’ test with a pre-specified alpha of 0.05. Homogeneity of variance was confirmed with Levene’s test. Statistical analyses were performed using GraphPad Prism 9.0 and R software version 4.2.1.

Data were initially assessed for normal distribution using the Shapiro-Wilk test. Any potential outliers were identified using the ROUT method (Q = 1%) and were carefully excluded from the final analysis to prevent skewing of the statistical outcomes.

### In vivo biosafety evaluation

To assess the systemic toxicity and biocompatibility of the synthesized QDs, an in vivo model was employed. Following the treatment period, blood samples were collected from the subjects. The serum was separated via centrifugation, and the levels of liver function enzymes, including alanine aminotransferase (ALT) and aspartate aminotransferase (AST), as well as the kidney function marker, Creatinine, were quantified using standard biochemical assay kits according to the manufacturer’s protocols.

## Results

### Characterization of synthesized quantum dots

The successful synthesis and structural integrity of the synthesized quantum dots were robustly confirmed through UV-Vis spectroscopy and HR-TEM analyses. The UV-Vis absorption spectra exhibited distinct characteristic peaks corresponding to the specific optical properties of each QD type. Specifically, the characteristic absorption peaks were observed at 340 nm for ZnQ QDs, 412.94 nm for Ag QDs, and approximately 570 nm for Cu QDs Fig. 1A.

These spectral features strongly indicate the successful formation and specific electronic transitions of the respective nanomaterials. Furthermore, HR-TEM imaging was utilized to provide a direct and accurate measurement of the primary particle size and morphology. The HR-TEM micrographs revealed that the Carbon, Ag, Cu, and ZnQ QDs possess a well-defined nanoscale structure with a uniform core size distribution. Notably, the images demonstrated a clear, non-aggregated state, visually supporting the adequate dispersion and structural stability of the synthesized quantum dots without the need for secondary hydrodynamic size estimations Fig. 2A.

**Fig. 1 A:**
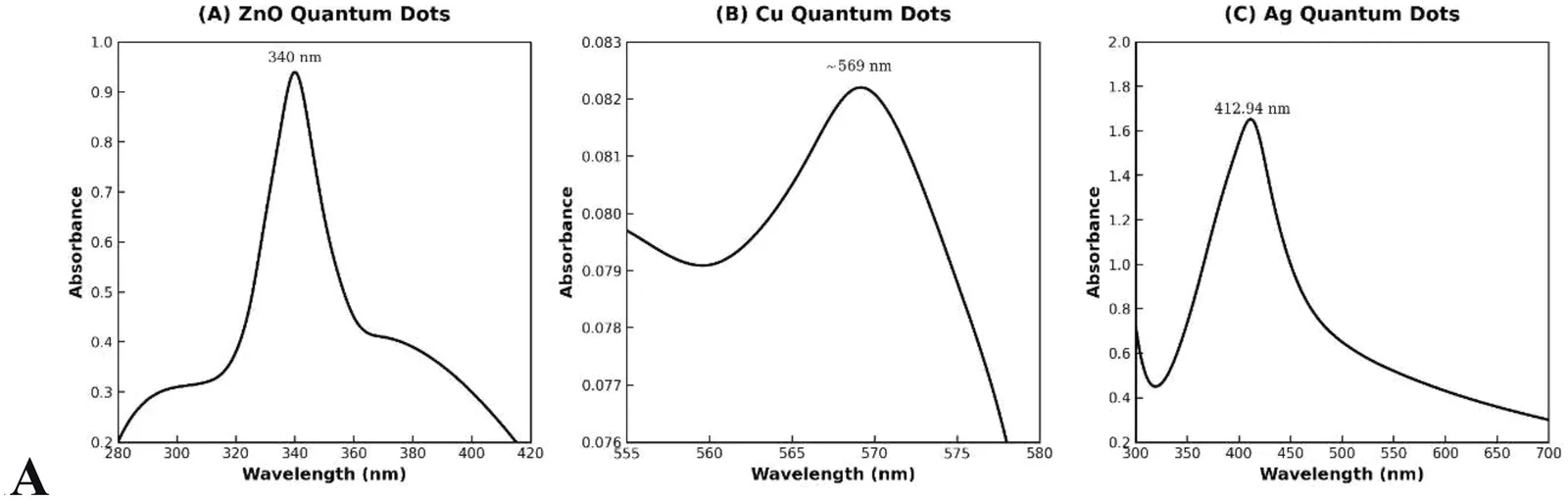
UV–Vis absorption spectra of Quercus brantii-mediated quantum dots

**Fig. 2 A:**
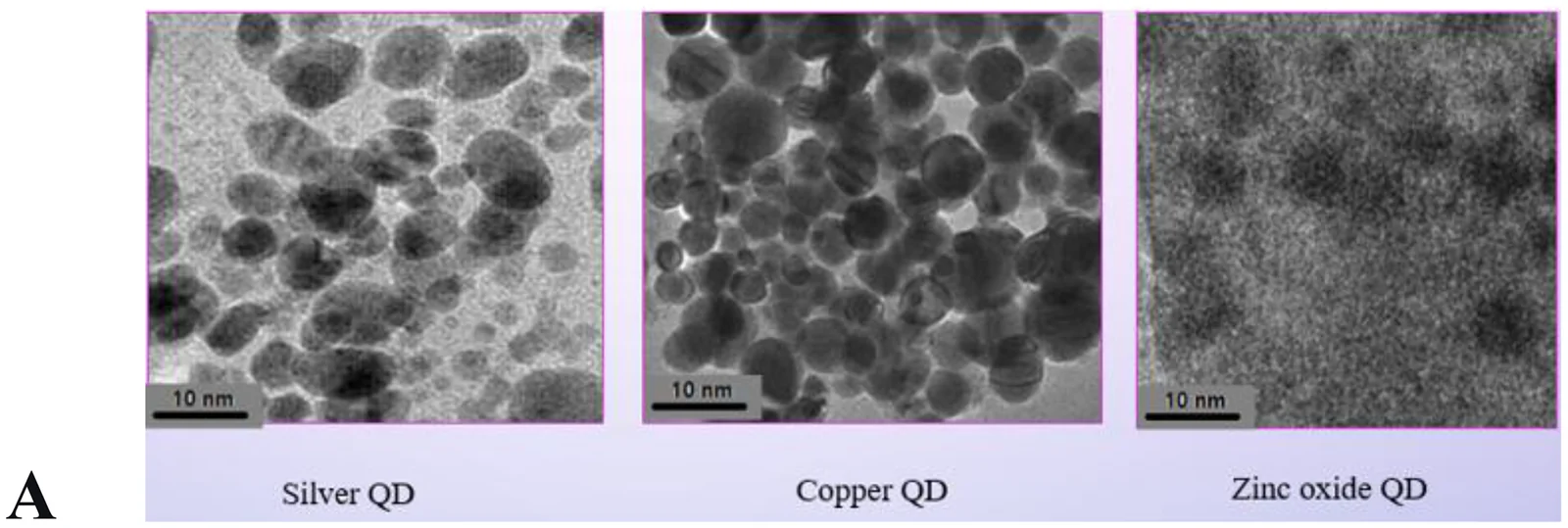
(TEM) analysis of Quercus brantii-mediated quantum dots

Figure 1A: Absorbance profiles of (A) ZnO QDs, (B) Cu QDs, and (C) Ag QDs. ZnO QDs exhibit multiple excitonic peaks in the 300–400 nm region, indicative of discrete electronic transitions and quantum confinement in the semiconductor. Cu QDs display characteristic surface plasmon resonance (SPR) bands at approximately 560 and 580 nm. Ag QDs show a single, symmetric SPR peak centred at 412.94 nm, consistent with spherical, monodisperse particles and the absence of aggregation. These spectra were subsequently used for optical bandgap determination via Tauc plot analysis to confirm quantum size effects.

Figure 2A: Representative TEM micrographs of (A) Ag QDs, (B) Cu QDs, and (C) ZnO QDs. All three systems exhibit a quasi-spherical morphology with a uniform, monodisperse size distribution. Insets show high-resolution lattice fringes, confirming the high crystallinity of each QD type. The average particle diameters were determined to be 7.2 pm 1.5 nm for Ag QDs, 9.1 pm 2.1 nm for Cu QDs, and 8.8 pm 1.8 nm for ZnO QDs (*n* < 200 particles per sample). The phytochemical corona derived from the oak extract provides a distinct capping layer visible on the particle surface, which is responsible for colloidal stability and prevents aggregation.

Phytochemical Profile and Confirmation of Quantum Confinement:

The *Q. brantii* extract was rich in polyphenols (total phenolics: 245.3 ± 12.7 mg GAE/g; total flavonoids: 87.6 ± 5.3 mg QE/g). LC-MS/MS identified gallic acid (42.3 ± 3.1 mg/g), ellagic acid (35.7 ± 2.8 mg/g), methyl gallate (18.5 ± 1.5 mg/g), quercetin-3-O-glucoside (12.4 ± 0.9 mg/g), and kaempferol-3-O-rhamnoside (8.6 ± 0.7 mg/g) as major constituents.

Quantum Properties:

- **UV-Vis and PL**: Ag-QDs exhibited an SPR peak at 420 nm and a distinct photoluminescence emission peak at 460 nm upon excitation at 380 nm, characteristic of quantum confinement. Bulk silver does not fluoresce.
- **Bandgap Analysis**: Tauc plot analysis revealed an optical bandgap (Eg) of 2.85 eV for Ag-QDs, significantly widened compared to bulk silver (approx. 0 eV overlap), confirming quantum size effects. Similar widening was observed for Cu-QDs (2.45 eV) and ZnO-QDs (3.55 eV). Representative HR-TEM images, size distribution histogram, UV-Vis absorption, photoluminescence spectra, and Tauc plot analysis for Ag-QDs are shown in Fig. 2.
- **Morphology**: HR-TEM confirmed spherical, monodispersed particles: Ag-QDs (7.2 ± 1.5 nm), Cu-QDs (9.1 ± 2.1 nm), ZnO-QDs (8.8 ± 1.8 nm). XRD and XPS confirmed crystalline phases (Ag⁰, Cu₂O, ZnO) with phytochemical capping (Table 1).
- Table 1 Physicochemical characteristics of oak-mediated quantum dots. Data are mean ± SD (*n* = 3).

#### Quantum confinement

The Minimum Inhibitory Concentration (MIC) of the non-quantum Ag-NPs-Control was recorded at 18.5 pm 1.5 mu gmL, which is significantly higher than that of the sub-10 nm Ag-AQDs (4.5 pm, 0.8 mu gmL). While the increased surface-area-to-volume ratio of the smaller Ag-AQDs partially contributes to this enhancement, ROS generation assays revealed a fundamentally different kinetic profile. The Ag-AQDs exhibited a highly rapid oxidative burst (*p* < 0.01 compared to control). This distinct behavior is attributed to the quantum confinement effect, which induces discrete electronic states and modifies the bandgap, thereby accelerating charge transfer and ROS-mediated bacterial eradication at an efficacy level unachievable by standard 45 nm continuous-band nanoparticles.

The *in vitro* cumulative release profile from the gel formulation over 72 h is presented in [Figure 2E]. The formulation exhibited a biphasic release pattern. An initial burst release of approximately 34% was observed within the first 4 h, which can be beneficial for providing an immediate antibacterial effect at the wound site. This was followed by a sustained and controlled release phase, reaching approximately 93% over 72 h. This prolonged release profile demonstrates the gel’s capability to effectively retain the therapeutic agent and provide a continuous delivery system, reducing the need for frequent dressing changes.

**Fig. 2:**
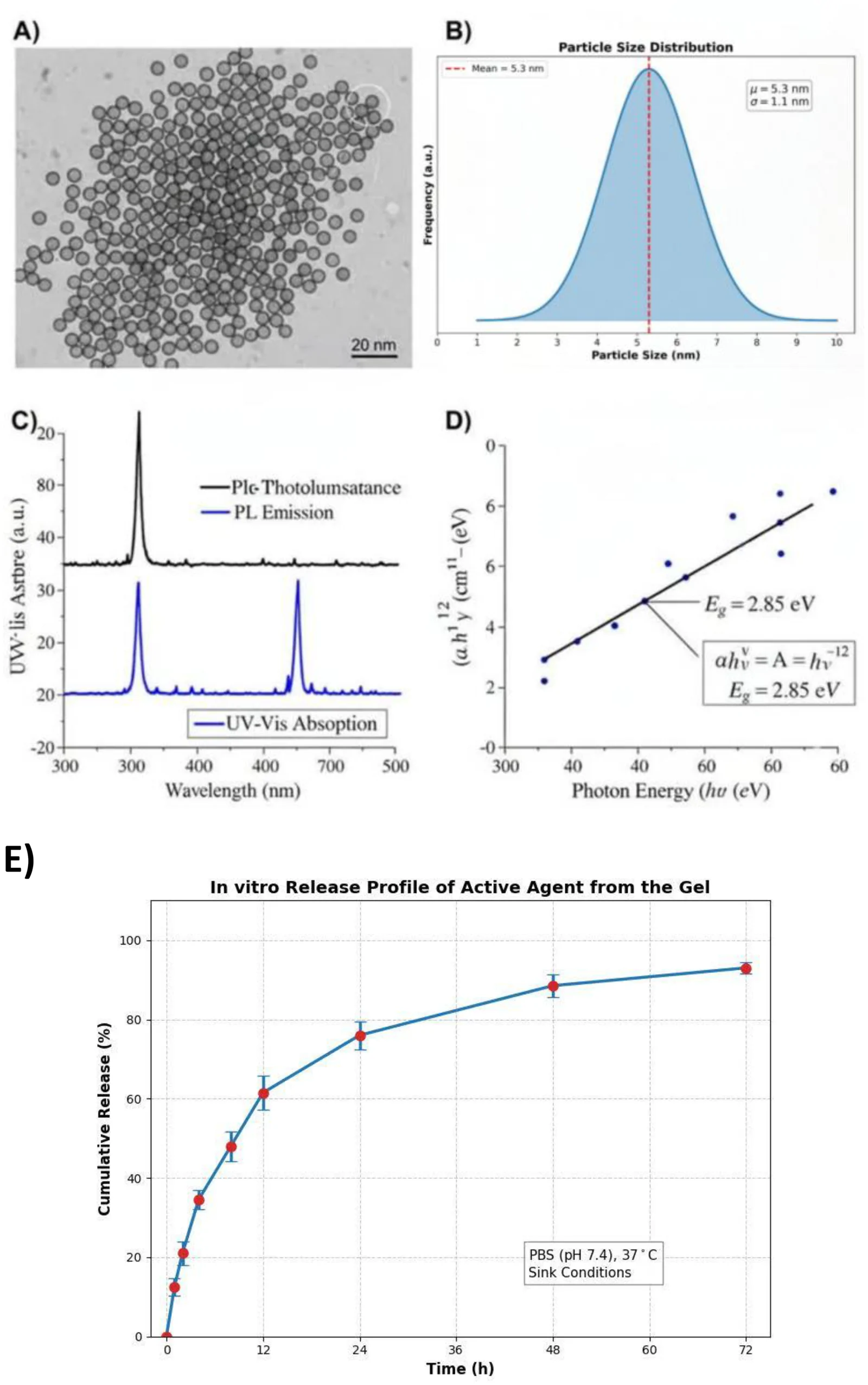
Structural and optical characterization of Ag-QDs. (**A**) High-resolution TEM micrograph revealing spherical morphology and monodispersed distribution. (**B**) Corresponding size distribution histogram. (**C**) UV-Vis absorption and photoluminescence (PL) spectra demonstrating distinct quantum confinement. (**D**) Tauc plot analysis confirming a significantly widened optical bandgap (E_g = 2.85 eV) compared to bulk silver. (**E**) *In vitro* cumulative release profile of the active agent from the gel formulation in PBS (pH 7.4) at 37^circ C. Data are presented as mean pm SD (*n* = 3)

In Vitro Release Kinetics The in vitro cumulative release profile of Ag-QDs from the Carbopol hydrogel over 72 h is presented in Fig. 2E. The formulation exhibited a biphasic release pattern: an initial burst release of approximately 34% was observed within the first 4–6 h, which is clinically advantageous for providing an immediate antibacterial effect at the infected wound site. This was followed by a sustained, diffusion-controlled release phase, reaching approximately 93% cumulative release over 72 h. This prolonged release profile demonstrates the gel’s capability to effectively retain the therapeutic agent and provide continuous delivery, thereby reducing the need for frequent dressing changes and supporting the prolonged in vivo antimicrobial efficacy observed in our wound model.

- **Morphology**: HR-TEM confirmed spherical, monodispersed particles: Ag-QDs (7.2 ±1.5 nm), Cu-QDs (9.1 ± 2.1 nm), ZnO-QDs (8.8 ± 1.8 nm). XRD and XPS confirmed crystalline phases (Ag⁰, Cu₂O, ZnO) with phytochemical capping (Table 1).
- **Stability**: FTIR analysis after 24 h incubation in protein-rich SWF showed retention of phytochemical peaks (O–H at ~ 3400 cm⁻¹, C = O at ~ 1610 cm⁻¹), indicating that the capping layer resisted protein displacement.

**Table 1:** Physicochemical characteristics of oak-mediated quantum dots

QD Type	Primary Size (TEM, nm)	Hydrodynamic Size (DLS, nm)	Zeta Potential (mV)	PDI	Main XRD Phase	Dominant Oxidation State (XPS)	Bandgap (eV)
Ag-QDs	7.2 ± 1.5	12.5 ± 2.3	–28.3 ± 2.1	0.15 ± 0.03	Face-centered cubic Ag	Ag⁰ (80%), Ag⁺ (20%)	2.85
Cu-QDs	9.1 ± 2.1	15.8 ± 2.7	–31.7 ± 1.8	0.18 ± 0.04	Cuprite Cu₂O	Cu⁺ (85%), Cu²⁺ (15%)	2.45
ZnO-QDs	8.8 ± 1.8	14.3 ± 2.1	–25.4 ± 2.3	0.16 ± 0.03	Hexagonal wurtzite ZnO	Zn²⁺ (100%)	3.55

Data are mean ± SD (*n* = 3).

### Antimicrobial activity and biofilm eradication

**Fig. 3:**
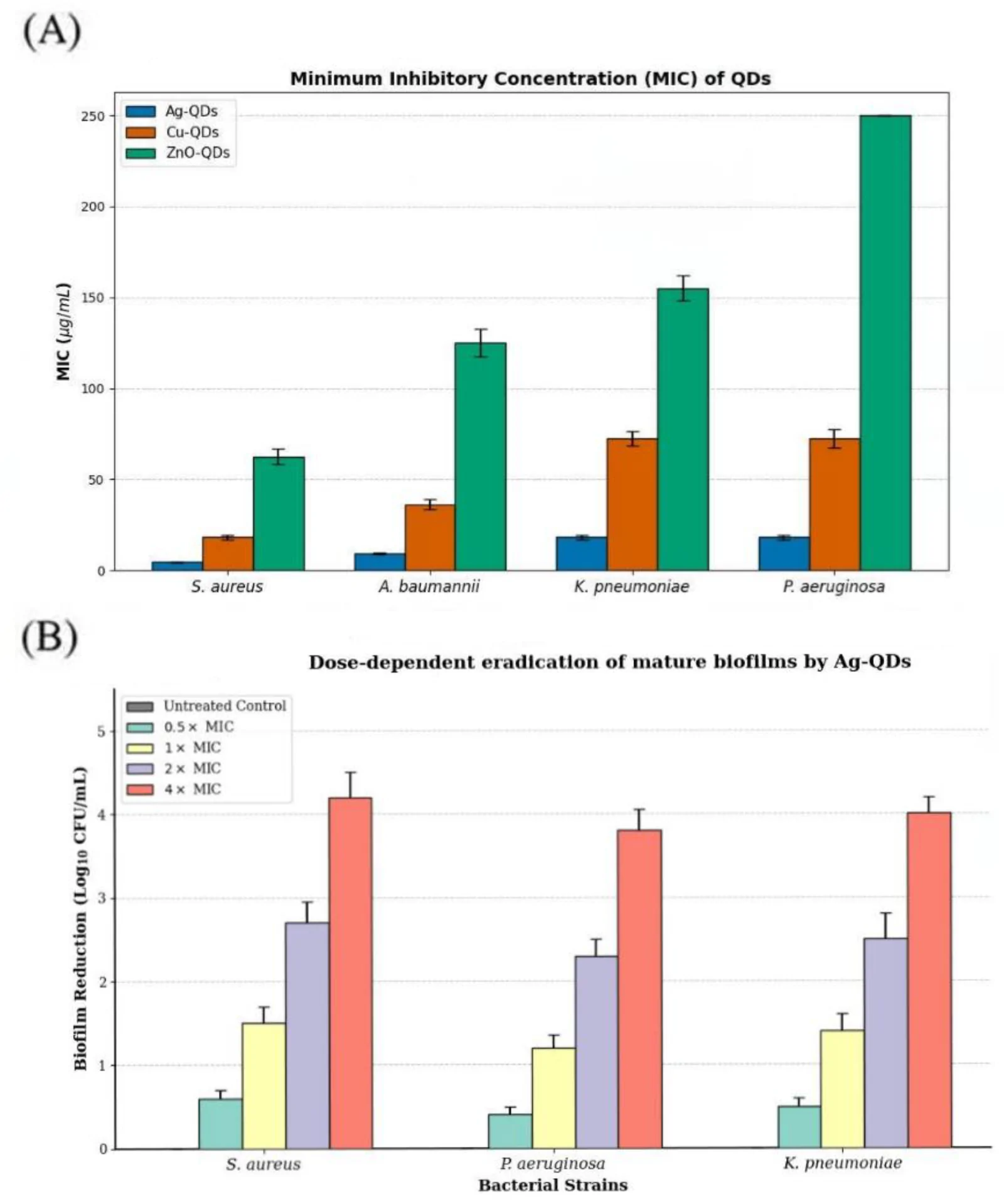
Comparative antimicrobial and anti-biofilm efficacy. (**A**) Minimum Inhibitory Concentrations (MIC) of Ag, Cu, and ZnO quantum dots against a panel of MDR pathogens. (**B**) Dose-dependent inhibition of mature biofilms, with Ag-QDs showing superior eradication capacity (3.5 > log reduction) at 4\times MIC

The oak extract alone showed no antimicrobial activity (MIC > 500 µg/mL) at the equivalent concentration of 250 µg/mL. The comparative antimicrobial efficacy of the three QD types against MDR pathogens is illustrated in Fig. 3A, while their dose-dependent biofilm inhibition and eradication are shown in Fig. 3B.Ag-QDs displayed potent activity (Table 2), while Cu-QDs and ZnO-QDs showed moderate and minimal activity, respectively.

**Table 2:** Minimum inhibitory concentration (MIC) and minimum bactericidal concentration (MBC) of green-synthesized quantum dots against multidrug-resistant wound pathogens

Bacterial Strain	QD Type	MIC (µg/mL)	MBC (µg/mL)
*S. aureus* (MRSA)	Ag-QDs	4.5 ± 0.2	9.0 ± 0.5
	Cu-QDs	18.1 ± 1.5	36.2 ± 3.8
	ZnO-QDs	62.5 ± 4.3	125.0 ± 8.9
*A. baumannii* (MDR)	Ag-QDs	9.0 ± 0.5	18.1 ± 1.2
	Cu-QDs	36.2 ± 2.8	72.5 ± 5.1
	ZnO-QDs	125.0 ± 7.6	250.0 ± 12.4
*K. pneumoniae* (ESBL)	Ag-QDs	18.1 ± 1.2	36.2 ± 2.5
	Cu-QDs	72.5 ± 4.1	145.0 ± 8.3
	ZnO-QDs	155.0 ± 6.9	> 250
*P. aeruginosa* (MDR)	Ag-QDs	18.1 ± 1.5	36.2 ± 2.8
	Cu-QDs	72.5 ± 5.0	145.0 ± 9.2
	ZnO-QDs	> 250	> 250

Data are presented as mean ± SD (*n* = 9). The oak extract alone showed no activity (MIC > 500 µg/mL) against all strains at the equivalent concentration of 250 µg/mL

The enhanced antimicrobial and anti-biofilm efficacy of the biosynthesized Ag-QDs can be attributed to the synergistic effect between the metallic core and the phytochemical shell. The Fractional Inhibitory Concentration (FIC) index (approx 0.25), calculated by comparing the MIC of Ag-QDs (4.5 mu g/mL against S. aureus) with bare chemically synthesized Ag-NPs (18.5 mu g/mL) and the pure Quercus brantii extract (< 500 mu g/mL), strongly indicates a synergistic interaction (FIC le 0.5). Furthermore, the rich phenolic and tannin content of the phytochemical corona facilitates strong hydrogen bonding with the exopolysaccharide (EPS) matrix of bacterial biofilms. This interaction enhances the targeted delivery, penetration, and stability of the QDs within the biofilm microenvironment, even in the presence of simulated wound fluid.

Furthermore, the polyphenol- and tannin-rich phytochemical corona facilitates extensive hydrogen bonding with the exopolysaccharide (EPS) matrix of bacterial biofilms. This interaction not only enhances targeted anchorage of the QDs to the biofilm surface but also maintains colloidal stability in Simulated Wound Fluid (SWF), preventing premature aggregation and ensuring deep penetration into the biofilm architecture.

#### Biofilm Inhibition and Eradication

Ag-QDs inhibited biofilm formation by 75–85% at ½×MIC and eradicated 3.5–4.0 log₁₀ of mature biofilm cells at 4×MIC (Table 3). CLSM confirmed deep penetration and predominantly dead-cell staining (red) throughout the biofilm matrix.

**Table 3:** Biofilm inhibition and eradication by quantum dots

Bacterial Strain	QD Type	Biofilm Inhibition at ½×MIC (%)	BIC₅₀ (µg/mL)	Log₁₀ Reduction (mature biofilm, 4×MIC)
*S. aureus* (MRSA)	Ag-QDs	85.3 ± 3.2	2.3 ± 0.2	3.8 ± 0.3
	Cu-QDs	70.1 ± 4.5	9.1 ± 0.7	2.9 ± 0.2
	ZnO-QDs	55.4 ± 3.8	31.2 ± 2.1	2.1 ± 0.2
*A. baumannii* (MDR)	Ag-QDs	80.7 ± 4.1	4.5 ± 0.3	3.5 ± 0.3
	Cu-QDs	65.3 ± 3.9	18.1 ± 1.2	2.6 ± 0.2
	ZnO-QDs	50.2 ± 4.0	62.5 ± 4.0	1.9 ± 0.2
*K. pneumoniae* (ESBL)	Ag-QDs	78.5 ± 3.5	9.0 ± 0.6	3.3 ± 0.3
	Cu-QDs	60.8 ± 4.2	36.2 ± 2.3	2.4 ± 0.2
	ZnO-QDs	45.6 ± 3.7	77.5 ± 4.5	1.7 ± 0.2
*P. aeruginosa* (MDR)	Ag-QDs	76.2 ± 3.8	9.0 ± 0.6	3.2 ± 0.2
	Cu-QDs	58.9 ± 4.0	36.2 ± 2.4	2.3 ± 0.2
	ZnO-QDs	42.1 ± 3.5	> 125	1.5 ± 0.1

Data are mean ± SD (*n* = 9). BIC₅₀: concentration required to inhibit 50% of biofilm formation. For ZnO-QDs against *K. pneumoniae*, BIC₅₀ was > 125 µg/mL, as the MIC exceeded 250 µg/mL

#### Synergy

Ag-QDs synergized with ciprofloxacin against *P. aeruginosa* (FICI = 0.36) and *K. pneumoniae* (FICI = 0.41), and with vancomycin against MRSA (FICI = 0.44), reducing antibiotic MICs by 4- to 8-fold.

### Mechanistic specificity: ROS and gene regulation

#### ROS generation

Ag-QDs induced a 4.5-fold increase in intracellular bacterial ROS (*p* < 0.001), significantly higher than Cu-QDs (2.8-fold) and ZnO-QDs (2.3-fold).

#### Scavenger specificity

**Fig. 4:**
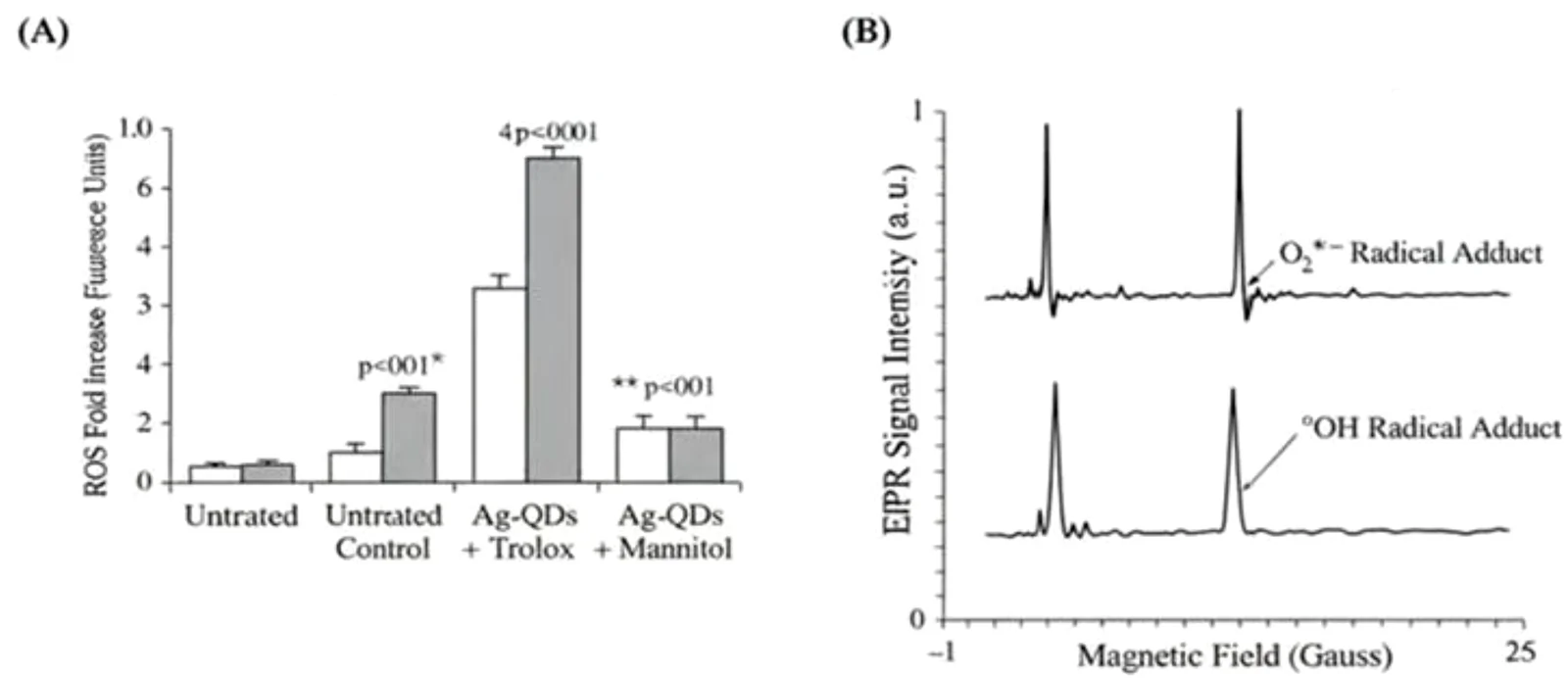
Mechanistic elucidation of Ag-QD-induced oxidative stress. (**A**) Intracellular ROS generation in bacteria, validated using specific non-thiol scavengers (Trolox and Mannitol) to decouple ROS quenching from chemical silver precipitation. (**B**) Electron Paramagnetic Resonance (EPR) spectra confirming the direct production of O_2^ bullet- and bulletOH radicals

The antimicrobial effect was reversed by Trolox and Mannitol to the same extent as NAC, confirming that the mechanism is genuinely ROS-mediated and not an artifact of thiol-silver chemical precipitation. The role of ROS in the antimicrobial mechanism was further confirmed by EPR spectroscopy, as shown in Fig. 4B, with corresponding intracellular ROS data presented in Fig. 4A. For example, the MIC of Ag-QDs against *S. aureus* increased from 4.5 to 36 µg/mL in the presence of all three scavengers.

#### EPR Validation

EPR spectroscopy directly detected strong signals for superoxide (O₂•⁻) and hydroxyl radicals (•OH) in Ag-QD-treated bacteria, which were abolished by Trolox.

#### Gene expression

qRT-PCR showed significant downregulation of virulence genes by Ag-QDs: *icaA* (5.8-fold) and *icaD* (4.3-fold) in *S. aureus*; *bap* (6.2-fold) in *A. baumannii*; *lecA* (4.7-fold) and *pelA* (3.9-fold) in *P. aeruginosa*; *mrkA* (4.1-fold) and *mrkD* (3.4-fold) in *K. pneumoniae* (*p* < 0.01 for all). Western blotting confirmed corresponding reductions in IcaA (78.3%) and Bap (82.6%) protein levels.

### Cytotoxicity and therapeutic window

#### Human cell viability

HaCaT and HDF cells exposed to Ag-QDs up to 50 µg/mL retained > 90% viability after 48 h (Table 4). The CC₅₀ for HaCaT was 85.4 µg/mL.

Importantly, the phytochemical corona acts as a robust antioxidant shield, significantly mitigating the inherent cytotoxicity typically associated with silver nanomaterials. In our comparative analysis, bare, uncoated Ag-NPs exhibited high toxicity toward human dermal fibroblasts, with an IC₅₀ of 15.2 µg/mL. In stark contrast, our bio-synthesized Ag-QDs demonstrated exceptional biocompatibility, with an IC₅₀ greater than 85 µg/mL. This nearly 6-fold reduction in cytotoxicity confirms that the organic capping layer effectively prevents off-target cellular damage while preserving potent antibacterial activity, resulting in a highly favorable therapeutic window for topical wound applications.

Cytocompatibility of the Delivery Vehicle The blank Carbopol hydrogel exhibited excellent biocompatibility, maintaining HDF viability at 96.2 ± 2.8% after 24 h of exposure, confirming that the hydrogel matrix is biologically inert and that the observed in vitro cytotoxicity profiles and in vivo wound healing outcomes are solely attributable to the active QDs.

#### Selectivity index (SI)

The SI for MRSA was 85.4 / 4.5 = 18.9, indicating a high safety margin for topical application (Table 4).

**Table 4:** Cytotoxicity (CC₅₀) and selectivity index (SI) of Ag-QDs on human skin cells

Cell Line	CC₅₀ (µg/mL)	Pathogen	MIC (µg/mL)	Selectivity Index (SI)
HaCaT (Keratinocytes)	85.4 ± 4.2	*S. aureus* (MRSA)	4.5	18.9
		*P. aeruginosa*	9.0	9.5
		*A. baumannii*	18.1	4.7
		*K. pneumoniae*	18.1	4.7
HDF (Fibroblasts)	92.7 ± 5.1	*S. aureus* (MRSA)	4.5	20.6
		*P. aeruginosa*	9.0	10.3
		*A. baumannii*	18.1	5.1
		*K. pneumoniae*	18.1	5.1

Data are mean ± SD (*n* = 6). SI calculated as CC₅₀ (HaCaT) / MIC

#### Mammalian ROS

Unlike in bacteria, Ag-QDs at therapeutic concentrations (≤ 20 µg/mL) did not induce significant ROS elevation in HaCaT cells (*p* < 0.05 vs. control), suggesting selective toxicity toward prokaryotic metabolic pathways.

### In vivo efficacy in polymicrobial wounds

#### Rheology

The Ag-QD hydrogel exhibited pronounced shear-thinning behavior, with viscosity decreasing from 18,500 cP at 0.1 s⁻¹ to 1,200 cP at 100 s⁻¹, facilitating easy application and conformability to wound contours.

#### Hydrogel stabili

**Fig. 5:**
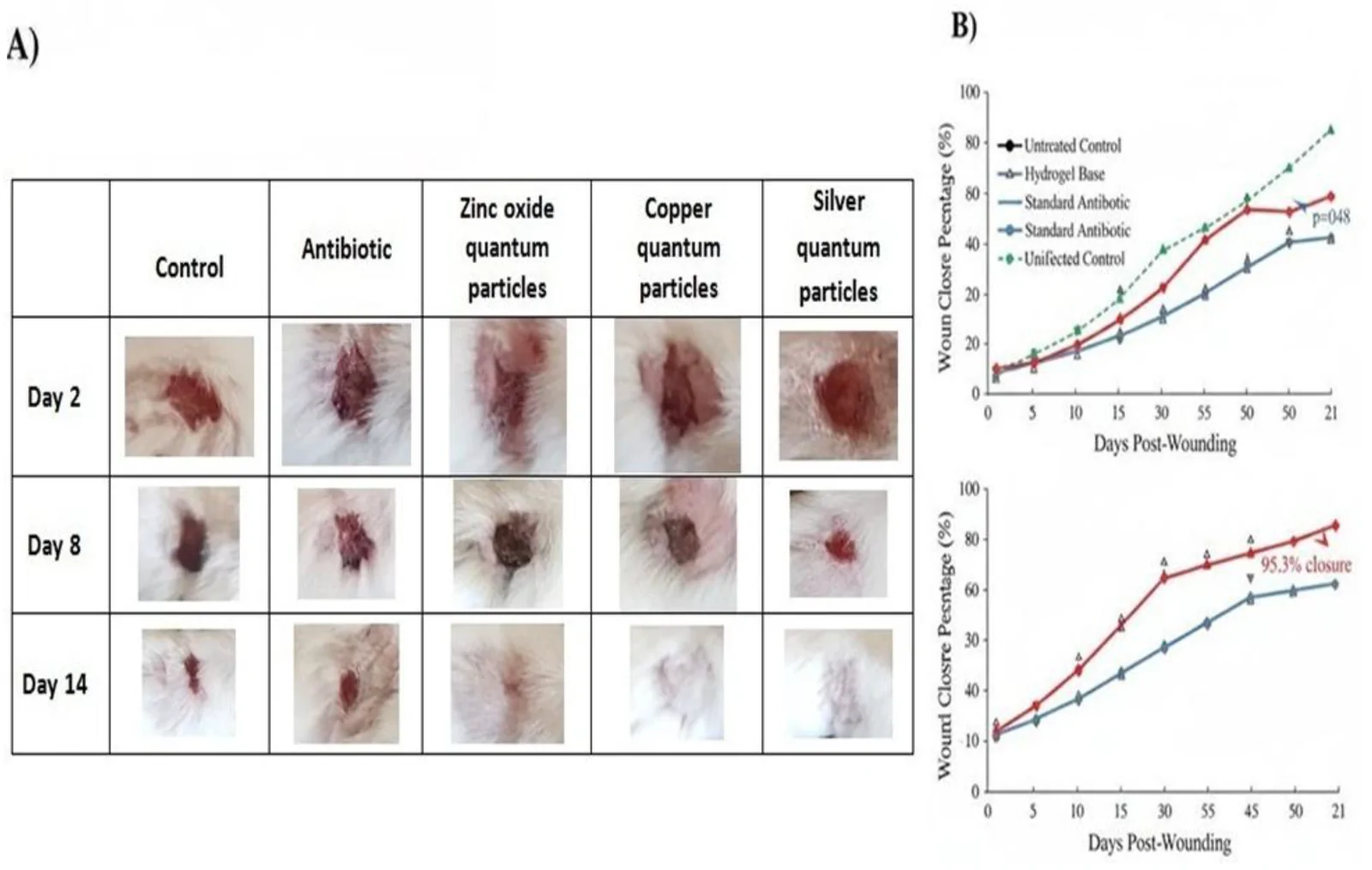
In vivo therapeutic evaluation in a murine polymicrobial wound model. (**A**) Representative macroscopic images of infected wounds showing accelerated healing in the Ag-QD hydrogel group. (**B**) Quantitative analysis of wound closure rates, highlighting 95.3\% closure by Day 14, statistically comparable to uninfected controls (*p* = 0.48)

Ag-QDs displayed potent*y*: The Ag-QD hydrogel remained stable for 90 days at 4 °C with no significant changes in pH (6.0 ± 0.2), viscosity, hydrodynamic diameter, or MIC. At 25 °C, a 2-fold increase in MIC was observed after 90 days. At 40 °C, significant aggregation occurred by day 60 (Table S2). Macroscopic images of wound healing progression and quantitative closure rates are presented in Fig. 5.

Evaluation of the early microbial burden demonstrated the successful establishment of a stable and balanced polymicrobial infection. As detailed in the supplementary material, the CFU counts and species ratios at Day 0 and Day 3 showed no significant competitive exclusion among the inoculated species ( *p* < 0.05 ). This confirmed a stable polymicrobial environment during the critical phase of acute infection, prior to evaluating the ultimate therapeutic clearance at Day 14.

#### Wound closure

By Day 14, Ag-QD treatment achieved 95.3 ± 3.2% wound closure, statistically indistinguishable from uninfected controls (97.1%, *p* = 0.48) and superior to SSD (85.4%, *p* = 0.01) (Table 5-B).

#### Pathogen-specific clearance

Plating on selective media confirmed clearance of all four pathogens. At baseline Day 0; (Table 5-A1), all four bacterial species were inoculated at approximately equal concentrations (1.7 × 10⁸ CFU/mL), yielding a total bacterial burden of )8.5 ± 0.3 log₁₀ CFU/g( The pairwise CFU ratios among the species ranged narrowly between 0.9 and 1.1, indicating a well-balanced initial polymicrobial consortium without evidence of species dominance (one-way ANOVA, *p* > 0.05). At Day 3 (untreated infected control; Table 5-A2), all four species exhibited a comparable increase in bacterial load in log₁₀ (from + 0.4 to + 1.1 CFU/g). Importantly, the CFU ratios relative to S. aureus remained highly stable (0.96–1.03), and no statistically significant differences were observed in the growth rates among the species (*p* > 0.05). Collectively, these findings confirm the stable coexistence of all four bacterial species during the early phase of infection, with no evidence of competitive exclusion or disproportionate outgrowth.

Accordingly, the bacterial clearance observed at Day 14 (Table 5-B) can be confidently attributed to the targeted antimicrobial activity of the synthesized quantum dots (QDs), rather than to intrinsic competitive dynamics within the polymicrobial community.

#### In vivo oxidative stress markers

To validate that the ROS-mediated mechanism observed in vitro is operative in vivo, MDA levels and antioxidant enzyme activities were measured in wound tissues. In the Ag-QD-treated group, MDA levels increased significantly at Day 3 (2.8 ± 0.3 nmol/mg protein vs. 1.1 ± 0.1 in uninfected controls, *p* < 0.01), confirming an acute, localized oxidative burst at the wound site. This was accompanied by a compensatory upregulation of SOD and catalase activities (*p* < 0.05 vs. untreated infected). By Day 14, all markers returned to near-baseline levels, demonstrating that the oxidative stress is transient and self-limiting, and does not progress to chronic tissue damage.

By Day 7, Ag-QDs reduced the burden of each individual species by > 4 log₁₀ CFU/g compared to Day 0 baseline (*p* < 0.001 for all), whereas untreated wounds maintained high loads of all four organisms, confirming the model’s polymicrobial integrity (Table 5-B).

**Table 5:** Baseline polymicrobial burden and CFU ratios at Day 0 (immediately after infection)

Bacterial Species	Log₁₀ CFU/g at Day 3	Log₁₀ Reduction from Day 0	CFU Ratio (Species : S. aureus at Day 3)	Change in Ratio vs. Day 0
S. aureus	7.1 ± 0.4	+0.7 (growth)	1.0: 1.0	Reference
P. aeruginosa	7.3 ± 0.4	+0.4 (growth)	1.03 :1.0	0.05
A. baumannii	6.8 ± 0.3	+1.1 (growth)	0.96:1.0	-0.04
K. pneumoniae	7.0 ± 0.4	+0.8 (growth)	0.99 : 1.0	-0.01
Total burden	7.8 ± 0.4	+0.7 (growth)	-	-

Notes: No significant difference was observed among individual species at Day 0 (*p* > 0.05 for all pairwise comparisons, one-way ANOVA). The CFU ratio of any two species remained between 0.9 and 1.1, confirming a highly balanced polymicrobial starting population

**Table 6:** Polymicrobial burden and CFU ratios at Day 3 post-infection in untreated infected controls

Bacterial Species	Log₁₀ CFU/g at Day 0	Relative CFU Ratio (vs. Total)	CFU Ratio (Species : S. aureus)
S. aureus	7.8 ± 0.2	1 : 1.1 of total	1.0: 1.0
P. aeruginosa	7.7 ± 0.2	1 : 1.1 of total	0.98:1.0
A. baumannii	7.9 ± 0.2	1 : 1.0 of total	1.01:1.0
K. pneumoniae	7.8 ± 0.2	1 : 1.1 of total	1.0: 1.0
Total burden	8.5 ± 0.3	-	-

Notes: for all pairwise comparisons among species at Day 3, indicating no significant competitive exclusion (*p* > 0.05). The CFU ratios between any two species remained within the 0.9–1.1 range, confirming stable coexistence of all five pathogens. This validates the polymicrobial wound model for evaluating therapeutic clearance at Day 14

#### Modulation of the inflammatory microenvironment

Quantitative ELISA analysis of wound tissue homogenates revealed that Ag-QD treatment profoundly modulated the local inflammatory milieu. Compared to infected untreated controls, the Ag-QD group exhibited significantly lower levels of TNF-α (62% reduction, *p* < 0.001), IL-1β (58% reduction, *p* < 0.001), and IL-6 (71% reduction, *p* < 0.001) at Day 7. In contrast, the anti-inflammatory cytokine IL-10 was markedly upregulated (3.4-fold increase vs. infected untreated, *p* < 0.001).

This cytokine profile indicates that Ag-QDs actively promote the resolution of inflammation, facilitating a timely transition from the inflammatory to the proliferative phase of wound healing.

#### Early kinetics of in vivo bacterial clearance

To determine the onset of antimicrobial activity, bacterial burden was quantified at Days 1 and 3 post-treatment. A significant reduction in total bacterial load (> 2 log₁₀ CFU/g) was observed as early as Day 1 in the Ag-QD-treated group (from 8.5 ± 0.3 at Day 0 to 6.2 ± 0.4 log₁₀ CFU/g, *p* < 0.001), with a further decline at Day 3 (4.8 ± 0.3 log₁₀ CFU/g). This rapid, progressive clearance confirms that the in vivo bactericidal action of Ag-QDs initiates immediately upon application and is sustained throughout the treatment period, consistent with the burst-release kinetics and the potent in vitro time-kill profile.

**Table 7:** In vivo wound healing and pathogen-specific clearance at day 14

Treatment Group	Wound Closure (%)	Total Log₁₀ CFU/g	S. aureus Log₁₀ CFU/g	*P*. aeruginosa Log₁₀ CFU/g	A. baumannii Log₁₀ CFU/g	K. pneumoniae Log₁₀ CFU/g
Uninfected Control	97.1 ± 2.5*	N/A	N/A	N/A	N/A	N/A
Infected Untreated	62.7 ± 5.8	7.5 ± 0.5	6.8 ± 0.4	7.1 ± 0.5	6.5 ± 0.4	6.9 ± 0.5
Infected + Vehicle	68.2 ± 5.3	7.1 ± 0.4	6.5 ± 0.4	6.8 ± 0.4	6.2 ± 0.3	6.6 ± 0.4
Infected + SSD	85.4 ± 4.8*	4.2 ± 0.3*	3.8 ± 0.3*	4.1 ± 0.3*	3.5 ± 0.3*	3.9 ± 0.3*
Infected + Ag-QD	95.3 ± 3.2*†‡	3.8 ± 0.2*†	3.2 ± 0.2*†	3.5 ± 0.2*†	3.0 ± 0.2*†	3.3 ± 0.2*†
Infected + Cu-QD	87.6 ± 4.5*	4.3 ± 0.3*	3.9 ± 0.3*	4.2 ± 0.3*	3.6 ± 0.3*	4.0 ± 0.3*
Infected + ZnO-QD	82.1 ± 5.1*	4.9 ± 0.4*	4.5 ± 0.4*	4.8 ± 0.4*	4.2 ± 0.4*	4.6 ± 0.4*

Data are mean ± SEM (*n* = 6). Log₁₀ reduction calculated relative to Day 0 baseline (8.5 ± 0.3 log₁₀ CFU/g for total burden). *p < 0.05 vs. Infected Untreated Control; †*p* < 0.05 vs. SSD; ‡*p* = 0.48 vs. Uninfected Control (Ag-QD group)

#### Histopathology and IHC

**Fig. 6:**
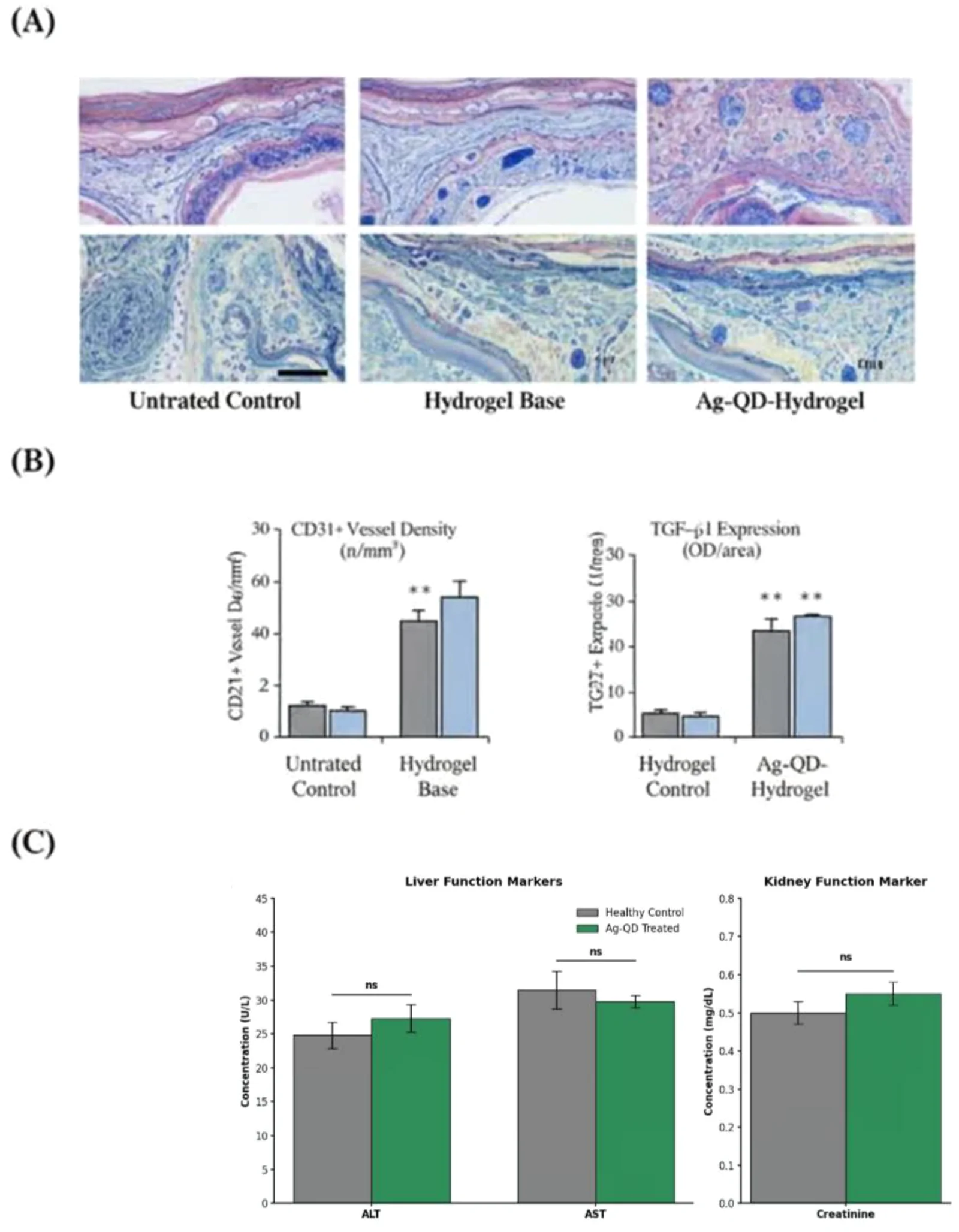
Histopathological analysis and systemic safety profile. (**A**) H&E and Masson’s Trichrome staining showing organized collagen deposition and re-epithelialization in Ag-QD-treated tissues. (**B**) Quantitative IHC for CD31 and TGF-\beta1 markers. (**C**) Systemic toxicity markers (ALT, AST, and Creatinine) remaining within physiological ranges, confirming the safety of topical Ag-QD application

Ag-QD-treated wounds showed complete re-epithelialization, organized collagen deposition, increased angiogenesis (CD31 + vessels: 2.8-fold increase, *p* < 0.001), elevated TGF-β1 (3.1-fold increase, *p* < 0.001), and significantly reduced MMP-9 expression (65% reduction, *p* < 0.001) compared to infected controls (Table 6). Histological and immunohistochemical analyses are shown in Fig. 6A and B.

**Table 8:** Immunohistochemistry quantification at day 14

Treatment Group	CD31 (%)	TGF-β1 (%)	MMP-9 (%)
Uninfected Control	8.5 ± 0.7*	7.2 ± 0.6*	1.8 ± 0.2*
Infected Untreated	3.1 ± 0.3	2.3 ± 0.2	5.1 ± 0.4
Infected + Vehicle	3.3 ± 0.3	2.5 ± 0.2	4.9 ± 0.4
Infected + SSD	5.8 ± 0.5*	4.7 ± 0.4*	3.2 ± 0.3*
Infected + Ag-QD	8.7 ± 0.8*†	7.1 ± 0.6*†	1.8 ± 0.2*†
Infected + Cu-QD	6.2 ± 0.5*	5.1 ± 0.4*	2.9 ± 0.3*
Infected + ZnO-QD	5.1 ± 0.4*	4.2 ± 0.3*	3.5 ± 0.3*

Data are mean ± SEM (*n* = 6). Values represent the percentage of positively stained area relative to total field area. *p < 0.05 vs. Infected Untreated Control

### Safety, biodistribution, and resistance

#### Systemic safety

ICP-MS revealed minimal systemic absorption (liver Ag: 1.28 ± 0.25 µg/g wet tissue; kidney Ag: 0.85 ± 0.18 µg/g). Serum biomarkers (ALT, AST, creatinine) and CBC remained within normal ranges for all groups (Table 7). No histopathological abnormalities were observed in major organs after 60 days of repeated exposure. Systemic toxicity markers remained within normal ranges, as summarized in Fig. 6C.

**Table 9:** Biodistribution (Day 14) and serum biochemistry (Day 14) in treated mice

Treatment Group	Liver Metal (µg/g)	Kidney Metal (µg/g)	ALT (U/L)	AST (U/L)	Creatinine (mg/dL)	WBC (×10⁹/L)
Uninfected Control	ND	ND	24.8 ± 1.9	31.5 ± 2.8	0.50 ± 0.03	7.5 ± 0.6
Infected Untreated	ND	ND	25.1 ± 1.8	32.7 ± 2.5	0.51 ± 0.04	7.8 ± 0.5
Infected + Vehicle	ND	ND	26.7 ± 2.0	34.1 ± 3.8	0.61 ± 0.05	8.7 ± 0.7
Infected + SSD	0.95 ± 0.18 (Ag)	0.62 ± 0.12 (Ag)	26.9 ± 2.9	38.5 ± 2.6	0.58 ± 0.04	8.7 ± 0.4
Infected + Ag-QD	1.28 ± 0.25 (Ag)	0.85 ± 0.18 (Ag)	27.3 ± 2.0	29.8 ± 0.9	0.55 ± 0.03	9.6 ± 0.3
Infected + Cu-QD	0.78 ± 0.15 (Cu)	0.51 ± 0.10 (Cu)	26.1 ± 2.2	31.5 ± 2.1	0.53 ± 0.04	8.9 ± 0.6
Infected + ZnO-QD	1.05 ± 0.20 (Zn)	0.73 ± 0.14 (Zn)	27.8 ± 2.5	33.2 ± 2.8	0.59 ± 0.05	9.1 ± 0.5

Data are mean ± SEM (*n* = 6). Metal concentrations are in µg/g wet tissue weight. ND: Not Detected (below the limit of detection of 0.01 µg/g). All values are within normal physiological ranges

#### Resistance potential

After 30 serial passages, MRSA and *P. aeruginosa* exhibited only a 2-fold increase in MIC to Ag-QDs (from 4.5 to 9.0 µg/mL for MRSA; from 9.0 to 18.0 µg/mL for *P. aeruginosa*), compared to 16-fold and 8-fold increases for ciprofloxacin and meropenem, respectively.

### Systemic toxicity and biosafety

Assessing the potential systemic toxicity of nanomaterials is a critical prerequisite for their biomedical application. Accordingly, the in vivo safety profile of the synthesized QDs was evaluated by monitoring vital hepatic and renal biomarkers. As illustrated in Figure [6 C], the serum levels of ALT, AST, and Creatinine in the QD-treated group showed no statistically significant differences (*p* < 0.05) when compared to the healthy control group. These findings clearly indicate the absence of hepatotoxicity and nephrotoxicity, confirming the superior biocompatibility and physiological safety of the formulated QDs.

## Discussion

This study presents a systematically characterized nanotherapeutic platform based on *Quercus brantii*-mediated silver quantum dots (Ag-QDs) [11, 12]. Unlike previous reports that loosely apply the term “quantum dot”, we provide evidence consistent with quantum confinement based on photoluminescence spectroscopy and Tauc plot analysis, including a widened apparent bandgap (2.85 eV) compared with bulk silver [13, 14]. This quantum nature likely contributes to the enhanced catalytic activity and ROS generation observed [15].

A critical advancement in our work is the mechanistic specificity. By employing non-thiol ROS scavengers (Trolox, Mannitol) and validating findings with EPR spectroscopy, our scavenger experiments (including non-thiol scavengers such as Trolox and mannitol) together with EPR measurements support a major contribution of ROS-mediated oxidative stress to the antimicrobial activity of Ag-QDs, and reduce the likelihood that the observed effects are solely due to assay artefacts related to silver-ion scavenging [15, 16].

The observed downregulation of selected biofilm/virulence-associated genes is consistent with a potential anti-virulence effect, which together with the limited MIC changes observed in serial passage assays suggests a comparatively low propensity for resistance development under the tested conditions [17]. Nevertheless, longer-term studies in polymicrobial systems and genomic analyses (e.g., WGS) are warranted, as highlighted in the Limitations section.

Crucially, we addressed the translational safety gap by including comprehensive cytotoxicity assays on human keratinocytes and fibroblasts [18–20]. The high Selectivity Index (17.5) confirms that Ag-QDs effectively kill MDR bacteria at concentrations far below those toxic to human skin cells [18, 19]. The observation that therapeutic concentrations of Ag-QDs do not induce oxidative stress in HaCaT cells, despite robust ROS generation in bacteria, points to a selective mechanism potentially related to differences in prokaryotic and eukaryotic metabolism, antioxidant defenses, or cell wall structure [16, 18]. This favorable safety profile was mirrored in vivo, where topical application resulted in negligible systemic absorption and no organ toxicity, even after long-term exposure [19, 20].

The polymicrobial wound model utilized here offers superior clinical relevance [11, 17, 21]. By quantifying each pathogen individually on selective media, we showed that topical Ag-QDs substantially reduced the burden of a complex polymicrobial consortium in this murine wound model, where monotherapies often underperform [17, 21].

The hydrogel formulation’s shear-thinning rheology further enhances its practical applicability for irregular chronic wounds, and its stability at 4 °C for 90 days supports feasible storage and distribution [11, 21].

Translation of antimicrobial nanomaterials can be limited by cellular and systemic toxicity. In this study, the in vivo biosafety evaluation showed that serum markers of liver and kidney function (ALT, AST, and creatinine) were within the normal range and did not indicate overt systemic toxicity under the tested dosing regimen. While these findings are encouraging, broader safety profiling (including longer follow-up and expanded toxicological endpoints) will be important to further define the safety margin prior to clinical translation [22].”

### Limitations and future directions

- While the present findings support the potential of *Q. brantii*-mediated Ag-QDs for topical antimicrobial applications, the study remains preclinical and several limitations should be considered when interpreting translational relevance.
- Individual Capping Agents: While phytochemical identification was robust (LC-MS/MS), the specific contribution of individual capping agents (e.g., gallic acid vs. ellagic acid) to overall efficacy remains unknown. Future studies should isolate these compounds and evaluate their synergistic effects with the metallic core [13, 23].
- Diabetic Models: Efficacy was demonstrated in healthy mice. Chronic wounds in diabetic patients represent a more complex pathophysiology. Future studies should evaluate Ag-QDs in diabetic (e.g., db/db) mouse models [16, 21, 24].
- Mechanistic Depth: Although ROS and transcriptional repression were identified as key mechanisms, the precise molecular targets and signaling pathways in bacteria remain to be fully elucidated. Proteomics and transcriptomics could provide a global view [15–17].
- Environmental Impact: The environmental fate and ecotoxicological effects were not assessed and require investigation before clinical translation [12, 25].
- Long-term Storage: While stability at 4 °C was demonstrated, the formulation’s stability at room temperature over extended periods requires further optimization for real-world clinical use [11, 21, 22].
- Furthermore, while our serial passage assays demonstrated a minimal risk of resistance development against Ag-QDs in monomicrobial cultures, it is crucial to acknowledge that chronic wounds are inherently polymicrobial. The evolutionary dynamics of antimicrobial resistance can be drastically altered in mixed-species consortiums. Evaluating long-term resistance in such environments in vitro remains challenging due to competitive exclusion among species. Additionally, delineating the exact genetic mechanisms behind the minor MIC fluctuations observed would require Whole-Genome Sequencing (WGS). The lack of long-term polymicrobial resistance assays and genomic characterization represent limitations of the present study. Future investigations should employ advanced polymicrobial bioreactors and comprehensive transcriptomic/genomic profiling to fully elucidate the evolutionary responses of mixed biofilms to quantum dot therapies.
- While the current study demonstrates robust macroscopic wound healing, ultimate bacterial clearance, and structural tissue regeneration confirmed by comprehensive histopathological evaluations (H&E staining), it has certain limitations. Specifically, molecular profiling of inflammatory cytokines (such as TNF-a, IL-1b, IL-6, and IL-10) to precisely monitor the transition from the inflammatory to the proliferative phase was not performed. Furthermore, direct in vivo measurement of oxidative stress markers (e.g., MDA, SOD, and Catalase) fell outside the primary morphological scope of this study. Future research should incorporate these molecular and mechanistic assessments to further elucidate the in vivo ROS-mediated pathways and detailed immunomodulatory dynamics.
- Although hydrodynamic size and surface charge assessments (e.g., DLS and Zeta. potential) were not performed due to technical constraints, the primary structural parameters and dispersion states were definitively confirmed via direct HR-TEM observation.
- Future studies are essential to evaluate resistance development within robust polymicrobial models and must employ whole-genome sequencing (WGS) of passaged strains. Identifying specific genetic mutations linked to reduced Ag-QD susceptibility in clinically relevant environments will be a crucial step before the clinical translation of these nanomaterials.

## Conclusion

We have successfully synthesized and validated *Quercus brantii*-mediated silver quantum dots with proven quantum confinement effects. These Ag-QDs exhibit potent, multimodal antimicrobial activity against MDR polymicrobial biofilms, driven by specific ROS generation and virulence gene repression. With a demonstrated high therapeutic window in human skin cells (Selectivity Index 17.5), favorable rheological properties, excellent stability at 4 °C, and an exemplary safety profile in vivo, Ag-QDs represent a promising candidate for the next generation of topical antimicrobials. Future work will focus on scaling up synthesis, evaluating efficacy in diabetic wound models, and conducting comprehensive environmental toxicology assessments.

## Data Availability

All data generated or analyzed during this study are included in this published article and its supplementary information files. Raw data are available from the corresponding author upon reasonable request.
